# Molecular Mechanisms of Curcumin in the Pathogenesis of Metabolic Dysfunction Associated Steatotic Liver Disease

**DOI:** 10.3390/nu15245053

**Published:** 2023-12-09

**Authors:** Marta Guariglia, Francesca Saba, Chiara Rosso, Elisabetta Bugianesi

**Affiliations:** Department of Medical Sciences, University of Turin, 10126 Turin, Italy; marta.guariglia@unito.it (M.G.); francesca.saba@unito.it (F.S.)

**Keywords:** curcumin, hepatic fibrosis, inflammation, insulin resistance, metabolic dysfunction associated steatotic liver disease, nutraceuticals, oxidative stress

## Abstract

Metabolic dysfunction-associated steatotic liver disease (MASLD) is a multifactorial condition characterized by insulin resistance, oxidative stress, chronic low-grade inflammation, and sometimes fibrosis. To date, no effective pharmacological therapy has been approved for the treatment of metabolic-associated steatohepatitis (MASH), the progressive form of MASLD. Recently, numerous in vitro and in vivo studies have described the efficacy of nutraceutical compounds in the diet has been tested. Among them, curcumin is the most widely used polyphenol in the diet showing potent anti-inflammatory and antifibrotic activities. This review aims to summarize the most important basic studies (in vitro and animal models studies), describing the molecular mechanisms by which curcumin acts in the context of MASLD, providing the rationale for its effective translational use in humans.

## 1. Introduction

Metabolic dysfunction associated fatty liver disease (MASLD) is one of the most common causes of liver disease, affecting 25% of the population, and its prevalence is increasing significantly in Western countries [1]. MASLD encompasses a spectrum of diseases characterized by hepatic steatosis when other causes of secondary hepatic fat accumulation (e.g., excessive alcohol consumption) cannot be identified. The etiology of MASLD is multifactorial; lifestyle, dietary habits, alterations in sugar and fat metabolism, and genetic predisposition contribute to the onset and progression of the disease. MASLD is often associated with the metabolic syndrome (hyperglycemia/type 2 diabetes mellitus [T2DM], hypertension, hypertriglyceridemia, low HDL cholesterol levels, and overweight/obesity) [1].

Hepatic steatosis, per se, is a benign and reversible condition, but, in some cases, it progresses to metabolic associated steatohepatitis (MASH), characterized by inflammation and ballooning degeneration [2]. The further progression of MASLD/MASH leads to hepatic fibrogenesis and ultimately cirrhosis, also contributing to the onset of hepatocellular carcinoma (HCC) [3]. MASLD is also associated with cardiovascular disease and T2DM-related complications such as nephropathy and neuropathy [4]. In this context, elucidating mechanisms of liver damage could unveil potential therapeutic targets for the management of patients with MASLD/MASH. In recent years, there has been an increasing interest in polyphenols, potent antioxidants contained in many foods, which can influence disease outcomes [5]; their consumption has been proven to ameliorate liver steatosis and to exert anti-fibrogenic effects in mice models of MASLD [6,7]. Curcuminoids, which include curcumin, are part of this group of compounds and have several health benefits, including anti-inflammatory, antioxidant, immunomodulatory, and antitumor properties [8]. The main issue related to the use of curcumin as nutraceutic in the clinical setting is its low bioavailability even through intraveneous administration routes. However, benefic effects of curcumin (prevention of oxidative stress, improvement of insulin resistance (IR), modulation of de novo lipogenesis) make it a potential therapeutic strategy able to prevent or improve metabolic derangements [9]. In this review, we will focus on basic studies (in vitro and animal models) in order to summarize the most important molecular mechanisms through which curcumin acts in the context of MASLD/MASH, and to encourage translational research in humans to validate the potential benefits of curcumin in patients with MASLD.

## 2. Pathophysiology of MASLD

The mechanisms involved in the pathogenesis of MASLD are complex and multifactorial; over the years, several theories have been proposed but they cannot fully describe the complexity of the disease. MASLD results from the combination of multiple environmental and genetic factors acting differently in individuals to provide a widely heterogeneous phenotype [10]. Many studies have shown that IR is the metabolic hallmark of MASLD. Specifically, IR is defined as the inability of cells to respond to the insulin stimulus in different tissues and organs. In the liver, IR leads to an increased endogenous glucose production with subsequent hyperglycemia followed by compensatory hyperinsulinism. In the muscle, IR leads to a decrease in glucose uptake by the myocytes resulting in hyperglycemia and, again, compensatory hyperinsulinism [11]. In the adipose tissue level, IR impairs the inhibition of lipolysis, contributing to the increased flow of free fatty acids (FFAs) to the liver. This FFA excess, along with increased de novo lipogenesis stimulated by hyperinsulism, leads to steatosis and triggers lipotoxicity [12]. The formation of toxic lipid species such as some ceramides can cause hepatocellular damage through endoplasmic reticulum (ER) stress, mitochondrial damage, inflammation, ballooning degeneration, and apoptosis [13]. These processes lead to an increased FFAs oxidation by mitochondria and reactive oxygen species (ROS) formation [14]. At high concentrations, ROS causes oxidative changes in cellular macromolecules (DNA, lipids, proteins, etc.) and leads to the accumulation of damaged compounds that predispose to liver injury [15]. The altered circulating milieu of cytokines in MASLD induces a state of low-grade chronic inflammation, enhances the nuclear transcription factor kappaβ (NF-κβ) signaling, and triggers a cascade of pro-inflammatory mediators such as tumor necrosis factor-alpha (TNF-α), interleukin-6 (IL-6) and interleukin-1 β (IL-1β). These cytokines are implicated in the activation and recruitment of Kupffer cells (KCs), which in turn mediate inflammation in MASH [16]. KCs are also involved in the pathogenesis of hepatic fibrosis, along with signals from stressed or damaged hepatocytes. All these signals lead to the activation of hepatic resident stellate cells (HSCs) into myofibroblasts that produce matrix proteins, thus promoting fibrogenesis [17].

Another important player in the pathogenesis of liver damage is the gut–liver axis. Over the last few years, increasing evidence suggests the importance of diet and gut microbiome in the onset and progression of MASLD [18,19,20]. Dysbiosis can compromise the intestinal barrier through the redistribution and disruption of tight junctions at the intestinal epithelium level, thus destroying paracellular transport and increasing intestinal permeability [21,22]. The latter allows the bacterial products, such as lipopolysaccharide (LPS), to enter the systemic circulation activating the TLR4 and the NF-κB pathway. Consequentially, the pro-inflammatory cascade exacerbates inflammation in the liver. Therefore, modulation of the gut microbiota represents a potential strategy for the prevention of MASLD mitigating chronic inflammation.

## 3. Nutraceutical Approaches in MASLD: Curcumin

In recent years, several therapeutic options have been proposed for the treatment of MASLD. However, no approved drugs for these patients are available yet, and clinical strategies are mainly based on diet, physical activity, lifestyle modification and control of the associated metabolic disorders [23,24]. Moreover, the use of probiotics, prebiotics, supplements or natural substances are also able to modulate the gut microbiota and to regulate the gut–liver axis [25,26,27]. Among these natural compounds, polyphenols are present in many foods and beverages and deserve attention for their antioxidant properties. Studies have shown that polyphenols can prevent oxidative stress, can promote FFAs oxidation, and can improve IR [28,29]. Furthermore, it has been reported that these compounds can modulate de novo lipogenesis by acting on the lipogenic enzymes and enhancing the expression of lipolytic proteins [30]. Curcumin is a lipophilic polyphenolic substance isolated from the rhizome of plants Zingiberaceae and Araceae through drying and grinding techniques. Curcumin is one of the main active constituents of Curcuma Longa, a common Asian spice of great pharmaceutical and commercial interest, used as a dietary spice, herbal remedy and in the beverage industry [31]. The use of curcuma has a thousand-year-old tradition; in Eastern cultures, it comes back over and over again, initially as a medicine, then as a cosmetic and spice, but it was also used in religious services [32,33]. In particular, in ancient Hindu medicine, it was used to treat sprains and swellings, while in Chinese medicine, it is used in the treatment of diseases related to abdominal pain. Its use is well documented in the treatment of various respiratory conditions (for example, asthma, bronchial hyperactivity and allergies), as well as for liver disorders, anorexia, rheumatism, diabetic wounds, cough and sinusitis [34]. Curcumin is mainly metabolized in the liver: the double bonds of curcumin are reduced to form dihydrocurcumin, tetrahydrocurcumin, hexa-drocurcumin and octahydrocurcumin. In addition, after oral ingestion, curcumin undergoes reduction, sulfation, and glucuronidation reactions in the liver, kidneys and the intestinal mucosa [35]. Diferuloylmethane (1,7-bis(4-hydroxy-3-methoxyphenyl)-1,6-heptadiene-3,5-dione), the alternative name of curcumin, has been shown to have biological activity in different cells through multiple molecular pathways [36]. However, due to its poor bioavailability, curcumin must be administered at high concentration through standardized supplements [36,37]. Many strategies are being studied to decrease its excretion, inhibit enzymatic reactions, or increase its availability and absorption by combining it with other elements. Piperine, for example, derived from black pepper, is an inhibitor of hepatic and intestinal glucuronidation of curcumin ensuring a high bioavailability of the compound [36,37].

An overview of molecular mechanisms in which curcumin is involved is summarized in Table 1. In the following paragraphs, we will thoroughly analyze the most important metabolic pathways targeted by curcumin in the context of MASLD. Animal models were used in most of the works. All mice were maintained under standard conditions of temperature and humidity, fed a steatogenic diet, and curcumin was administered orally or by injection at different concentration to explore its effects on different pathways.

### 3.1. Curcumin Improves Insulin Resistance

The oral administration of curcumin can ameliorate IR by improving glucose disposal and insulin sensitivity [38,39,64]. In the study by Shao et al., curcumin supplementation was able to improve glucose disposal in both the liver and adipose tissue of mice fed with a high-fat diet (HFD) [38]. Through the in vivo intraperitoneal glucose tolerance test, the authors demonstrated that curcumin administration reduced glucose increase improving insulin sensitivity. Moreover, in vitro experiments on the hepatoma cell line (HepG2) showed that curcumin dose-dependently restored the stimulatory effect of insulin also in the presence of glucose oxidase, a known inductor of oxidative stress [38]. Another study by Wang L. and colleagues demonstrated how the oral administration of curcumin in HFD-fed mice was able to inhibit adipose tissue lipolysis through the downregulation of CD36 expression; this led to a decreased flux of FFAs toward the liver and to hepatic IR improvement [39]. Further, by treating hepatocytes with conditioned medium from adipose tissue, the authors demonstrated how curcumin improved insulin signaling through the inhibition of diacylglycerol (DAG)-associated Protein kinase C (PKCε) pathway and preserved insulin action by inhibiting the glucose-6-phosphate (G6P)-α and phosphoenolpyruvate carboxykinase 1 (PCK1) pathways, thus suppressing gluconeogenesis [39].

### 3.2. Curcumin Reduces Oxidative Stress

Another important mechanism that can be targeted by curcumin is oxidative stress [15]. Vizzuti et al. showed that curcumin treatment was able to reduce the expression of the monocyte chemoattractant protein 1 (MCP-1) in mice exposed to a methionine/choline-deficient (MCD) diet. This effect was also associated with a reduction in oxidative stress assessed by oxidative changes in hepatocyte DNA, and with an improvement of necroinflammation. Moreover, the authors demonstrated that curcumin reduced ROS generation by HSCs through the inhibition of the expression of the metallopeptidase inhibitor type 1 (TIMP-1), which is triggered by oxidative stress [40]. Subsequently, Li B. and colleagues showed that in rats fed with a HFD, curcumin modulated the nuclear factor-erythroid 2-related factor-2 (Nrf2) pathway [41,65]. In particular, Nrf2 improved IR by increasing the expression of several Nrf2-related genes encoding for glutathione peroxidase (GSH), glutathione synthase (GS), kininine oxidase-reductase 1 (HO-1), glutamate cysteine ligase and many other factors [42]. Afrin R. et al. further analyzed the effects of curcumin on the progression of MASH in a mouse model of HCC and found that the biological compound was able to attenuate oxidative stress by decreasing the hepatic expression of cytochrome P450 family 2 subfamily E member 1 (CYP2E1) and CCAAT/enhancer binding proteins (C/EBPβ) [42]. Specifically, CYP2E1 activation is involved in FFAs metabolism and leads to the production of ROS. In this model of HCC-NASH, oxidative stress could be also improved through the attenuation of the Nrf2 pathway, as previously reported [42]. Once activated by oxidative stress, Nrf2 translocates into the nucleus where it binds to specific antioxidant transcription elements enhancing the expression of some antioxidant and detoxification genes such as HO-1, glutamate-cysteine ligase (GCLC), NAD(P)H dehydrogenase and quinone (NQO-1), which are well-characterized Nrf2-dependent antioxidant defense genes [42]. Xie Y. et al. demonstrated that pretreatment with curcumin in rats with acute liver injury (ALI) induced by lipopolysaccharide (LPS)/d-galactosamine (d-GalN), promoted Nrf2 nuclear translocation in a dose-dependent manner, amplifying the antioxidant response by enhancing hepatic expression of HO-1. These results demonstrated that protein levels of HO-1, GCLC, NAD(P)H and NQO-1 increased in liver tissues after LPS/GaLN injection, particularly when rats were treated with curcumin [43]. The authors also focused on malondialdehyde (MDA), which is the end product of lipid peroxidation, as well as an excellent marker of oxidative stress; the liver tissues of ALI-induced rats were characterized by a high expression of MDA, which decreased in the subgroup of animals pretreated with curcumin [43]. The improvement of antioxidant systems by curcumin has been also described by Cunnigham R. and colleagues [44]. In particular, the authors showed that in female Wistar rats, curcumin was able to increase the hepatic expression of superoxide dismutase 1 (SOD1), one of the main systems involved in the elimination of free superoxide radicals [44]. Lee DE. et al. showed that in the liver tissue of C57BL/6J mice, curcumin administration was able to induce the expression of sirtuin 1 (SIRT1), another factor that is part of the antioxidant systems [45]. After proteomic analysis of the liver of mice fed with MCD or curcumin-supplemented MCD, the authors found two differentially expressed patterns of proteins; eight of these were known to undergo O-GlcNAcylation modification, an event that is upregulated in MASH mice through the hexosamine biosynthetic pathway (HBP), which regulates the endoplasmic reticulum (ER) stress responses. Finally, the authors demonstrated that curcumin was able to decrease the intracellular ROS levels induced by palmitate treatment, suggesting its potential protective effect against toxic lipid species [45].

Another target of curcumin is the peroxisome proliferator-activated receptor alpha (PPAR-α) that regulates the upstream signaling pathways of the autophagy AMP-activated protein kinase (AMPK) and Phosphoinositide 3-kinases (PI3K)/serine/threonine kinase 1 (AKT)/Mammalian target of rapamycin (mTOR), leading to an increase in the autophagic flow into the hepatocytes. Interestingly, this mechanism seems to inhibit the production of the extracellular matrix (ECM), preventing hepatic fibrosis [46].

### 3.3. Curcumin Modulates Lipid Metabolism

Oxidative stress, through the generation of ROS, induces lipid peroxidation, destroying unsaturated FFAs in the cell membranes, causing a decrease in endogenous antioxidants, thus resulting in liver tissue damage. Recently, Mahmouda A. et al. showed that curcumin decreased MDA levels and restored glutathione peroxidase (GPx) activity in the liver tissue of MASH mice [47]. There is a close relationship between the dysregulation of lipid homeostasis and oxidative stress, as ROS production induced by fat and by pro-inflammatory cytokines storm contributes to the onset and progression of MASLD [66]. For example, the antioxidant factor Nrf2 may be involved in the reduction in lipids levels and the attenuation of liver damage. In the study by Yan C. et al., the use of curcumin on mice fed with high-fat and high-fructose diets (HFHFr) alleviated the accumulation of lipid droplets in the liver through the inhibition of FFAs synthesis [48]. Moreover, the authors showed that curcumin dramatically upregulated the hepatic expression of CYP7A1 (one of the endogenous metabolism enzymes involved in the rate-limiting bile acids synthesis) and CYP3A (the most important hepatic cytochrome P450) through the regulation of Nrf2/FXR/LXRα pathway, enhancing the metabolic capacity and preventing/ameliorating MASLD [48]. Several studies on animal models treated with HFD described the beneficial effect of curcumin in lowering lipid levels and improving hepatic steatosis by interfering with the carbohydrate response element binding protein (ChREBP) and the sterol regulatory element-binding protein 1 (SREBP1-c) pathways in the liver. ChREBP and SREBP1-c are two important key transcriptional factors that stimulate the expression of lipogenic genes; their deregulation in the liver has been associated with the amount of hepatic steatosis [38,39,45,48,49,65]. Another protein that regulates hepatic energy metabolism and lipid homeostasis is AMP-activated protein kinase (AMPK). AMPK activation suppresses fatty acid and cholesterol biosynthesis by inhibiting the activity of the enzymes acetyl CoA carboxylase (ACC) and 3-hydroxy-3-methyl glutaryl CoA (HMG-CoA) reductase [67,68]. In addition, AMPK can inactivate SREBPs factors, thus inhibiting downstream lipogenic genes [69]. A study performed on HepG2 cells treated with oleic acid plus/minus curcumin demonstrated that the natural compound was able to reduce lipid accumulation through the activation of AMPK and the inhibition of SREBP-1c, thus reducing lipogenesis in the hepatocytes [50]. All this evidence suggests that the AMPK pathway may be targeted by natural compounds such as curcumin for the prevention and improvement of MASLD [70]. Another study by Zhao NJ. et al., describes a double and opposite effect of curcumin on lipid homeostasis through the Notch signaling in the liver [49]. Specifically, the authors found that curcumin was able to block Notch signaling in the liver of rats fed with HFD who developed hepatic steatosis, by the up-regulation of PPARα, (thus increasing FFAs oxidation), and by the down-regulation of lipogenic genes such as FAS and ACC (thus promoting lipids accumulation and hepatic steatosis development and worsening) [49]. Recently, theracurcumin, a more bioavailable synthetic form of curcumin, has been proven to reduce cholesterol levels by targeting both the HMG-CoA reductase and acyl-CoA: cholesterol acyltransferase (ACAT), two enzymes involved in cholesterol synthesis and esterification, respectively [51]. Niemann-pick C1-like 1 (NPC1L1) is a factor that mediates cholesterol absorption playing an important role in the pathogenesis of MASLD [71]. Yang J. et al. 2023 [52] showed that curcumin was able to protect HFD-induced MASLD by inhibiting both intestinal and hepatic NPC1L1 expression through the down-regulation of SREBP-2/hepatocyte nuclear factor-1 alpha (HNF1α) pathway, thus reducing intestinal cholesterol absorption and hepatic cholesterol accumulation improving liver steatosis and providing evidence for curcumin as a potential nutritional therapy in patients with MASLD [52].

### 3.4. Curcumin Ameliorates Inflammation

An important mechanism contributing to the pathogenesis of hepatic steatosis, and especially to the progression of steatohepatitis, is inflammation. In some of the studies analyzed in this paragraph, the anti-inflammatory role of curcumin in the modulation of the nuclear factor kappa B (NF-κB) pathway has emerged [42,45,49,72]. NF-κB represents a family of inducible transcription factors that regulate a wide range of genes involved in different processes of the immune and inflammatory response [20]. In the study by Afrin R. et al., curcumin reduced the progression of steatohepatitis in a mouse model of HCC-NASH by inhibiting HMGB1-NF-κB nuclear translocation. This event depends on the interaction between HMGB1 and the Toll-like receptor 4 (TLR4), and leads to the release of pro-inflammatory cytokines. In mice treated with curcumin, this pathway was inhibited and inflammation was improved [42]. The attenuation of inflammation by curcumin may also act through the inhibition of inhibitors of NF-κB (IkBs), as described by Xie L. et al. in a mouse model of LPS/D-D-GalN-induced ALI [43]. Curcumin may inhibit the NF-κB pathway also through the modulation of Notch-1. In mice fed with a HFD, the Notch-1 pathway is upregulated compared to normal controls, whereas curcumin treatment reverses this effect in a dose-dependent manner [49]. Similarly, Lee et al. observed the inhibitory effect of curcumin on NF-κB pathway in a mouse model of NASH through inhibition of O-GlcNAcylation [45]. Feng et al. showed that curcumin was able to downregulate TLR4, by inhibiting its interaction with the myeloid differentiation factor 88 (MyD88), thus blocking the translocation of the complex to the nucleus [53]. Conversely, in another study on female Wistar rats treated with different diet types, curcumin did not modulate markers of NF-κB signaling, despite reduced histological inflammation in treated groups [44]. Moreover, the authors demonstrated that curcumin administration decreased the expression of the SPP1 gene encoding for the osteopontin protein, which is involved in the progression of MASLD by generating a feedback loop between different inflammatory cytokines [44]. Specifically, curcumin may improve steatohepatis by reducing hepatic cell necrosis and by inhibiting the M1 polarization of macrophages thus reducing inflammatory factors secretion [54]. Finally, it has been recently demonstrated that curcumin treatment inhibits hepatic inflammation (TNF-α, IL-1β, Intercellular adhesion molecule-1 (ICAM-1), and vascular cell adhesion molecule 1(VCAM-1)) and angiogenesis (NO, endothelin-1 (ET-1), and angiopoietin 2 (ANGPT2)) by restoring the physiology of hepatic endothelial function via NF-κB and PI3K/Akt/hypoxia-inducible factor 1 (HIF-1α) signaling pathway modulation [55].

### 3.5. Curcumin Modulates Gut Microbiota Preventing MASLD

Over the last years, several studies focused on the interaction between curcumin and gut microbiota, identifying a bidirectional relationship. On one side, curcumin promotes the development of beneficial bacteria, improves gut barrier functions, and counteracts the expression of pro-inflammatory mediators. On the other hand, the gut microbiota, through specific metabolic pathways, metabolizes curcumin leading to the release of anti-inflammatory, antioxidant, and anti-tumor metabolites, thus exerting beneficial effects [73]. In vivo studies based on mice models in which MASLD was induced either with drugs or with diets have observed how curcumin can improve altered intestinal permeability by increasing the levels of tight junction proteins, such as zonulin-1 and occludin. Specifically, curcumin seems to modulate directly the intestinal flora by decreasing the levels of lipopolysaccharides (LPS), diamine peroxidase (DAO), and D-lactate [56,57,58]. LPS stimulates the production of pro-inflammatory cytokines causing IR, thus its modulation by curcumin prevents further progression of MASLD [57]. Li and colleagues, treated mice fed with HFD both with curcumin and metformin to assess changes in metabolic parameters such as body weight, biochemical parameters, livers status at histology and gut microbiota composition. Through next generation sequencing approach, the authors showed that both treatments were able to improve the Firmicutes/Bacteroides ratio. Specifically, they showed that in mice treated with curcumin the abundance of *Butyricicoccus* increased while the abundance of *Dorea* decreased paving the basis for a better understanding of the beneficial effects of specific bacterial species in the setting of MASLD [58]. A similar effect was also observed in the study by Li et al., where in curcumin-treated steatotic mice, both the Firmicutes/Bacteroidetes ratio [56] and Desulfovibrio endotoxin production were reduced, thus improving metabolic pathways such as oxidative phosphorylation, FFAs metabolism, glycolysis/gluconeogenesis, and biliary secretion compared to untreated mice [59].

### 3.6. Curcumin Exerts Antifibrotic Effects

The anti-fibrotic property of curcumin is less clear, but several lines of evidence show that it is able to stimulate the immune system, modulate inflammatory pathways, enhance the expression of pro/anti-apoptotic proteins, and mitigate oxidative stress, thus exerting anti-fibrotic effects. Curcumin exterts its anti-fibrogenic effects through the modification of HSCs, which differentiate into myofibroblasts able to synthetize collagen. In the study by Vizzuti et al., 2010, the antifibrogenic effect of curcumin was assessed in a mice fed with a MCD diet. Specifically, the addition of curcumin to the diet decreased the expression of both TIMP-1 and a-smooth muscle-actin (α-SMA) (two important factors involved in the process of fibrogenesis) in a dose-dependent manner [40]. Furthermore, the decrease in α-SMA was associated with a reduction in the expression of type I pro-collagen [40]. Treatment with curcumin inhibits the activation of insulin-dependent HSCs, interrupting the signal and suppressing the expression of the insulin receptor gene in Ito’s cells. Another anti-fibrogenic mechanism involves low-density lipoproteins (LDLs) and the property of curcumin to avoid oxidation, another factor able to stimulate the activation of HSCs [74]. The effect of curcumin on HSCs may also affect apoptosis by reducing MyD88 protein expression and by inhibiting the levels of the effectors involved in its metabolic pathways, such as TLR2, TLR4, Nf-kB, TNF-α and IL-1β [75]. The results of Huang’s study show that curcumin significantly reduces the expression of transforming growth-β1 (TGF-β1) and intrahepatic MCP-1 by preventing monocyte infiltration into the injured liver of mice treated with carbon tetrachloride (CCl4) [76]. Curcumin may also reverse the process of hepatic fibrogenesis by activating the PPAR-γ pathway, thus blocking the signaling through the platelet-derived growth factor subunit B (PDGF-b) and its receptors in the liver [60]. Moreover, curcumin can induce the expression of Nrf2 in primary HSCs via in vivo enhancing fibrogenesis [61,62]. Hepatic fibrogenesis may be also the result of an interaction between different organs and districts. It is well known how adipose tissue may influence hepatic inflammation and oxidative stress, thus enhancing HSCs differentiation and collagen deposition [77]. For example, leptin may activate HSCs promoting liver fibrogenesis; curcumin is able to eliminate the pro-fibrogenic effect of this hormone by interrupting the leptin signaling pathway [63]. All these data stimulate translational research in humans to confirm the use of curcumin in attenuating/improving liver fibrosis in subjects with MASLD.

### 3.7. Curcumin Supplementation for the Treatment of MASLD: An Update on Human Studies

In recent years, several clinical trials have investigated the beneficial effects of curcumin on MASLD patients focusing on the improvement of hepatic steatosis and fibrosis. For example, analyses of liver ultrasound scans showed an improvement in the degree of severity of MASLD in steatotic patients treated with curcumin (500 mg) and black pepper [78]. This improvement was also observed in the study by Saeede Saadati et al. after the administration of 1500 mg curcumin [79]. In the double-blind, placebo-controlled randomized trial by Mirhafez SR et al., 80 patients with MASLD, according to ultrasound examination and laboratory results, were treated with 250 mg/day of curcumin phytosome powder (equivalent to 50 mg/day pure curcuminoids) for two months. At the end of the intervention, the degree of hepatic steatosis and serum aspartate aminotransferase (AST) levels decreased significantly in the treated group compared with controls [80]. Safari et al. also observed in their patients with MASLD, a significant decrease in liver stiffness by Fibroscan and hepatic steatosis following the administration of 250 mg daily of curcumin [81]. Moreover, in a randomized placebo-controlled trial on 55 MASLD patients, Saberi-Karimiana et al. observed that capsules containing 500 mg curcumin were able to improve the levels of some inflammatory cytokines such as TNF-α, MCP-1 and epidermal growth factor (EGF) [8]. The beneficial effect of curcumin on the gut microbiota has also been tested in humans. In the study by Chashmniam et al., it was found that in MASLD patients, 250 mg of curcumin was able to decrease the serum levels of some metabolites, which were related to amino acids, bile acids (BAs), tricarboxylic acid (TCA) cycle and endotoxins produced by the microbiota, confirming its beneficial action [82]. However, the main issue concerning the use of curcumin as nutraceutic is the poor bioavailability both at the plasma and tissue level, because of its poor absorption, rapid metabolism, and fast systemic clearance. Consequently, over the years, efforts have been made to find new strategies for the administration of curcumin, such as the use of adjuvants, complexed/encapsulated formulas, and nanoparticles, so that its beneficial potential can be maximized [83].

## 4. Conclusions

**Figure 1:**
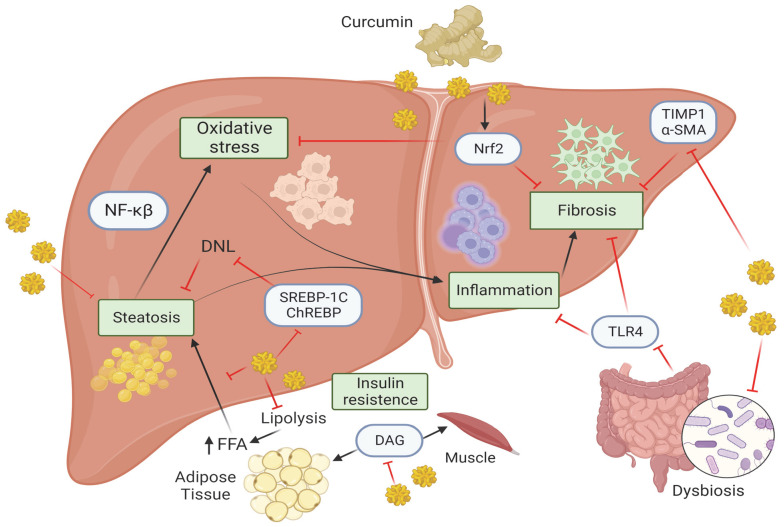
Molecular mechanism of curcumin in MASLD, Created with BioRender.com (Accesed on 1 October 2023). Curcumin, by acting on several molecular pathways, is able to attenuate the pathological processes that lead to the progression of MASLD. Curcumin reduces the accumulation of DAG in the muscle and adipose tissue thus affecting hepatic IR. Curcumin inhibits lipolysis thereby preventing the release of FFA from adipose tissue and preventing their accumulation in the liver (steatosis). The latter is also prevented by the inhibition of several factors such as ChREBP, SREBP1c, thus reducing DNL pathway. Hepatic steatosis enhances oxidative stress which is kept in check by curcumin through inhibition of Nrf2 pathway, which also acts on fibrosis. MASH is characterized by the presence of inflammation that in turn activates NF-κB pathway, which is inhibited by curcumin. Moreover, the gut microbiota can be regulated by curcumin through the inhibition of TLR4 signaling and consequently preventing inflammation and fibrosis. The latter is kept in check by curcumin through the inhibition of Nrf2, TIMP1 and α-SMA. Abbreviations: α-SMA, α-Smooth muscle actin; ChREBP, carbohydrate response element binding protein; DAG, diacyl glycerol; DNL, de novo lipogenesis; FFAs, free fatty acids; NF-κβ, Nuclear factor kappa β; NRF2, nuclear factor erythroid 2-related factor 2; SREBP1c, sterol regulatory element binding protein 1c; TIMP-1, metallopeptidase inhibitor type 1; TLR4, Toll-like receptor 4.

Curcumin is an important polyphenol, a naturally occurring bioactive compound that plays an important role in human nutrition. The potential benefit of curcumin, as shown in Figure 1, is related to its ability to improve IR, reduce oxidative stress, mitigate inflammation, exert antifibrotic effect, and restore eubiosis. So far, most of the evidence relies on animal and in vitro studies, but the consistent results encourages translational research in humans to confirm the potential benefits of curcumin in patients with MASLD. The novel technologies, including the use of spheroids or liver organoids, together with the integration of omics approaches, may be useful to understand the mechanistic role of curcumin in the context of hepatic fibrosis or HCC, thus improving our knowledge of the tangled mechanisms of liver damage and help to tailor new effective and safety treatments.

## Figures and Tables

**Table 1:** Overview of the studies describing the biological role of curcumin. This table shows all metabolic pathways (insulin resistance, oxidative stress, lipid metabolism, inflammation, gut microbiota, and hepatic fibrogenesis) and the physiological effects that curcumin exerts both in animal models than in vitro.

Animal/In Vitro Models	Physiological Effects	Dose of Curcumin	Duration of Treatment	Reference
Insuline resistence				
Animal model: C57BL/6J mice	Improvement of glucose levels	4 g/kg	2 days/week	Shao W et al., 2012 [38]
Animal model: C57BL/6 mice	Downregulation of CD36 expression. Decrease in flux of FFAs. Inhibition of DAG-PKCε and G6P-α and PCK1 pathway	50 mg kg^−1^	10 days	Wang L et al., 2016 [39]
Animal model: C57BL/6 mice In Vitro: HSCs	Inhibition the secretion of TIMP-1, MCP-1 and α-SMA	25 μg 20 μM	Weekly intervals for 10 weeks 30 min	Vizzutti F et al., 2010 [40]
Animal model: Sprague Dawley rats	Increase in GSH, GS, HO-1 and glutamate cysteine ligase. Deregulation of ChREBP and SREBP1-c	50 mg/kg	6 weeks	Li B et al., 2016 [41]
Animal model: C57BL/6J mice	Decrease in CYP2E1 and C/EBPβ. Attenuation of Nrf2. Inhibition of HMGB1-NF-κB translocation	100 mg/kg/day	4 weeks	Afrin R et al., 2017 [42]
Animal model: Sprague Dawley rats	Nrf2 traslocation. Increase in HO-1, GCLC, NAD(P)H and NQO-1. Decrease in MDA. Inibition of IkBs. Reduce of NF-κB activation	30, 60, 120 mg/kg	3 days	Xie YL et al., 2017 [43]
Animal model: Female Wistar rats	Increase in SOD1.	~100 mg/kg of body weight per day	8 weeks	Cunningham RP et al., 2018 [44]
Animal model: C57BL/6J mice In Vitro: AML12 cells	Induce of SIRT1. Decrease in ROS. Deregulation of ChREBP and SREBP1-c. Inhibition of O-GlcNAcylation and NF-κB	100 mg/kg 0.3, 3 μM	3 weeks 12 h	Lee DE et al., 2019 [45]
Animal model: Sprague Dawley rats In Vitro: BNL CL.2 cells	Inhibition of the EMT procession	100, 200, 400 mg/kg 10, 20, 30 μM/L	8 weeks 24 h	Kong D et al., 2020 [46]
Animal model: Male albino Wistar rats	Decrease in MDA and increase in GPx	60 mg/kg	16 weeks	Mahmouda, Ahmed M.M et al., 2021 [47]
Lipid metabolism				
Animal model: C57BL/6 mice In Vitro: Primary liver cells	Upregulation of CYP7A1 and CYP3A through the regulation of Nrf2/FXR/LXRα pathway	50, 100 mg/kg 10 μM	4 weeks 24 h	Yan C et al., 2018 [48]
Animal model: Male Sprague Dawley rats	Upregolation of PPAR-α. Downregulation of ACC and FAS. Downregulation of Notch signaling and NF-κB	100, 200 mg/kg/day	8 weeks	Zhao NJ et al., 2018 [49]
In Vitro: HepG2 cells	Reduction in AMPK and the inhibition of SREBP-1c	1, 5, 10, 25, 50 µM	24 h	Kang OH et al., 2013 [50]
Animal model: C57BL/6N mice	Targeting HMG-CoA and ACAT	theracurmin (500, 1000, 2000 mg/kg) curcumin (150, 300, 600 mg/kg)	12 weeks	Wang, J.W et al., 2019 [51]
Animal model: Male Syrian Golden Hamsters In Vitro: Caco-2; HepG2 cells	Downregulation of SREBP-2/HNF1α pathway	0.1% *w*/*w*	12 weeks 24 h	Yang, J et al., 2023 [52]
Inflammation				
Animal model: ApoE−/− mice with a C57/BL6 genetic background	Downregulation of TLR4 and NF-κB. Up-regulation of ZO-1 and occludin	0.1% *w*/*w*	16 weeks	Feng D et al., 2019 [53]
Animal model: Male C57BL/6 mice In Vitro: RAW264.7 cells	Reduciton of IL-1β, TNF-α and M1 macrophages	100 mg/kg 0, 2.5, 5, 10 μM	8 weeks 3 h	Tong C et al., 2021 [54]
Animal model: Sprague Dawley male rats In Vitro: LSECs	Modulation of NF-κB and PI3K/Akt/HIF-1α pathway	25, 50, 100 mg/kg 1, 2, 4, 8, 10 μM	8 weeks 24 h	Wu J et al., 2023 [55]
Gut microbiota				
Animal model: CD-1 male mice	Restoration of ZO-1 and occluding. Reduction in TLR4/NF-κB.	0.1% *w*/*w*	24 weeks	Hong T et al., 2022 [56]
Animal model: Sprague Dawley male rats	Restoration of ZO-1 and occluding. Regulation of LPS-binding protein and TNFα. Suppression of NF-κB and TLR4 up-regulation	200 mg/kg	12 weeks	Feng W et al., 2017 [57]
Animal model: Sprague Dawley male rats	Reduction in the Firmicutes/ Bacteroidetes ratio	200 mg/kg/day	14 weeks	Li R et al., 2021 [58]
Animal model: Male C57BL/6 mice	Reduction in the Firmicutes/Bacteroidetes ratio and desulfovibrio bacteria. Enrichment of oxidative phosphorylation, FFAs metabolism, glycolysis/gluconeogenesis, and biliary secretion	1.2 g	10 weeks	Li, S et al., 2021 [59]
Hepatic fibrogenesis				
In Vitro: HSCs	Enhancement of PPARγ activity. Inhibition of PDGF-b	20 μM	24 h	Lin J et al., 2008 [60]
Animal model: ICR mice In Vitro: Human LX-2 cells	Expression of Nrf2	100, 200, 400 mg/kg 10, 20, 40 μM	4 weeks 24 h	Lu C et al., 2017 [61]
Animal model: Albino Sprague Dawley	Expression of Nrf2	50 mg/kg/day	16 weeks	Abd El-Hameed NM et al., 2021 [62]
In Vitro: HSCs	Interruption of leptin signaling pathway	100 ng/m	24 h	Tang Y et al., 2009 [63]

Abbreviations: α-SMA, α-Smooth muscle actin; ACAT, acetoacetyl-CoA Thiolase; ACC, acetyl-CoA carboxylase; AKT, serine/threonine kinase 1; AML12, alpha mouse liver 12 cells; AMPK, AMP-activated protein kinase; BNL CL.2, embryonic liver cell line; C/EBPβ, CCAAT/enhancer binding proteins; CD36, cluster of differentiation; ChREBP, carbohydrate response element binding protein; CYP2E1, cytochrome P450 2E1; CYP3A, cytochrome P450 3A; CYP7A, cholesterol 7 alpha-hydroxylase; DAG-PKCε, diacylglycerol-protein kinase C; EMT, epithelial–mesenchymal transition; FAS, fatty acid synthase; FFAs, free fatty acids; FXR, farnesoid X receptor; G6P-α, glucose-6-phosphate dehydrogenase; GCLC, glutamate-cysteine ligase; GPx, glutathione peroxidase; GS, glutathione synthase; GSH, glutathione; HepG2, human hepatoma cells; HIF-1, hypoxia-inducible factor-1; HMG-CoA, hydroxymethylglutaryl-CoA synthase; HMGB1-NF-κB, high mobility group box 1- nuclear factor kappa B; HNF1α, hepatocyte nuclear factor 1 homeobox alpha; HO-1, heme oxygenase-1; HSCs, hepatic stellate cells; ICR, imprinting control region; IkBs, inhibitors of NF-κB; IL-1β, interleukin-1β; LPS, lipopolysaccharides; LSECs, human liver sinusoidal endothelial cells; LXRs, liver X receptors; MCP-1, monocyte chemoattractant protein-1; MDA, malondialdehyde; NAD(P)H, nicotinamide adenine dinucleotide phosphate; NQO1, NAD(P)H quinone oxidoreductase 1; NRF2, nuclear factor erythroid 2-related factor 2; PCK1, phosphoenolpyruvate carboxykinase 1; PI3K, phosphoinositide 3-kinases; PDGF-B, platelet-derived growth factor B; PPAR-α, peroxisome proliferator activated receptor alpha; ROS, reactive oxygen species; SIRT1, silent information regulator sirtuin 1; SOD1, superoxide dismutase 1 gene; SREBP1c, sterol regulatory element binding protein 1c; TIMP-1, metallopeptidase inhibitor type 1; TLR4, Toll-like receptor 4; TNF, tumour necrosis factor; ZO-1, zonulin. Curcumin concentration in animal models has been reported as g/kg, mg/kg·min, μg/kg or based on animal weight (0.1% *w*/*w* [weight/weight]); for in vitro models, the dosage of curcumi used is reported in μM, μM/L or ng/m.

## Data Availability

Data are contained within the article.
